# Intercalation of the anticancer drug lenalidomide into montmorillonite for bioavailability improvement: a computational study

**DOI:** 10.1007/s00894-024-06210-w

**Published:** 2024-12-04

**Authors:** Yumeida V. Meruvia-Rojas, Esther Molina-Montes, Alfonso Hernández-Laguna, C. Ignacio Sainz-Díaz

**Affiliations:** 1 Andalusian Earth Sciences Institute, CSIC, Av. de Las Palmeras, 4, 18100 Armilla Granada, Spain; 2 Faculty of Pharmacy, University of Granada, Granada, Spain; 3 Instituto de Investigación Biosanitaria, Ibs.GRANADA, Granada, Spain

**Keywords:** Lenalidomide, Multiple myeloma, Smectite, Intercalation, Crystal structure, Polymorphism, DFT, Force field, Cancer

## Abstract

**Context:**

Lenalidomide (LEN) is used for the treatment of myeloma blood cancer disease. It has become one of the most efficient drugs to halt this disease. LEN is a low-soluble drug in aqueous media. The search of a pharmaceutical preparation to improve the bioavailability and, therefore, to optimize its efficiency is an important issue for pharmaceutical industries and health care. The use of natural excipients such as montmorillonite (MNT) can provide changes in the physical–chemical properties for improving the bioavailability of this drug. We present the first computational study at the atomic scale of the periodic crystal forms of the polymorphs for this anticancer drug, highly demanded in the pharmacy market. In addition, we propose a pharmaceutical preparation by intercalation of LEN in natural MNT. So, our calculations predict that LEN can be intercalated in the interlayer space of MNT, and be released in aqueous media, and physiological aqueous media in consequence. This release process is a more exothermic reaction than the unpacking energy of any of its polymorphs. Besides, the infrared spectra of the LEN molecule and its crystal polymorphs, and LEN intercalated in the confined space of MNT, have been calculated at different levels of theory. The band frequencies have been assigned, matching with the experimental bands, predicting the use of this technique for experimental studies.

**Method:**

In this work, the method is aimed to explore this research at the atomic and molecular level by using computational modelling methods including INTERFACE FF and other FF along with quantum mechanical calculations (Dmol^3^ and CASTEP) of 3-D periodical systems applying periodical boundary conditions. Models of the isolated molecule and two polymorphs of the crystal structures, with the model of bulk water and LEN intercalated in the MNT model, have been considered. An analysis of the intermolecular interactions is accomplished.

**Supplementary Information:**

The online version contains supplementary material available at 10.1007/s00894-024-06210-w.

## Introduction

**Fig. 1:**
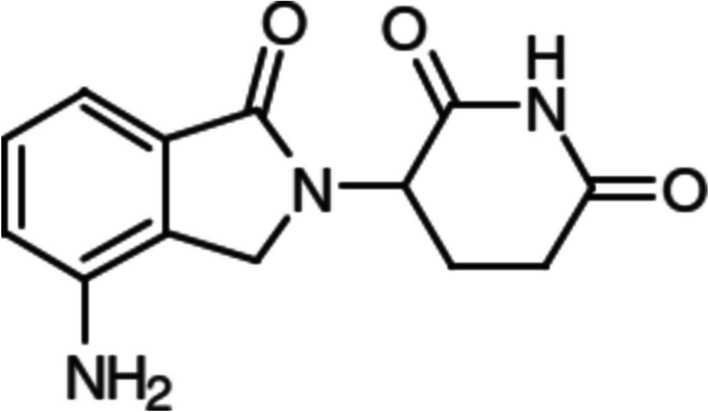
Lenalidomide [(RS)-3-(4-amino-1-oxoisoindolin-2-yl)-piperidine-2,6-dione]

Lenalidomide (LEN), (RS)-3-(4-amino-1-oxoisoindolin-2-yl)-piperidine-2,6-dione (Fig. 1), is an immunomodulatory imide drug (IMID) for anticancer treatment that has reached spectacular results for multiple myeloma (MM) disease, becoming a revolutionizing haematological cancer treatment [1]. MM is a type of cancer that involves the proliferation of abnormal plasma cells, which accumulate in the bone marrow. The growth of these malignant cells leads to bone weakening, anemia and alteration of the immune system response. Moreover, MM is characterized for releasing monoclonal immunoglobulin from malignant plasma cells into blood. IMIDs directly inhibit MM cell growth in the bone marrow microenvironment and promote effector immune cell function as well [2, 3]. More specifically, this drug binds the E3 ligase substrate adaptor CRBN (cereblon) to degrade IKZF1 (zinc finger region of protein) and the casein kinase 1α (CK1α), thereby leading to cell cycle arrest and apoptosis in MM. Also, the drug is capable of stimulating the immune system by activating T cells (CD4+ and CD8+), downregulating PD-1 receptor on the T cells. However, despite significant medical advances in recent years, including the use of these drugs in clinical settings, MM is still considered an incurable disease.

According to GLOBOCAN [4, 5] estimates, in 2020, there were 176,404 newly diagnosed MM cases in the world. Overall, MM is the second or third most common haematological malignancy, with an incidence ranging from 2.9 to 4.8 cases per 100,000 individuals per year. In Europe and the USA, 50,918 and 32,119 new cases, respectively, have been counted. The estimated number of deaths worldwide in 2020 was 117,077, with low-income countries showing an incidence/mortality ratio close to 1, as also reflected by a low survival rate [6]. In contrast, Europe and the USA exhibit a lower mortality rate. The use of LEN for its treatment probably explains much of the increasing survival. LEN has a recognized effectiveness and safety profile before and after bone marrow transplantation [7]; furthermore, LEN is also used as a maintenance treatment drug in order to minimize the risk of MM relapse [8]. Thus, in recent years, there has been a high demand for LEN, in an attempt to enhance overall survival in MM patients. LEN was the second IMID drug in revenues of the US pharmaceutical market with more than 6.660 million € in 2017. In 2022, Sandoz announced the launching of this drug in Europe, which is indicated for several haemato-oncology conditions as recommended by the European Society for Medical Oncology (ESMO) guidelines.

In MM, various combinations of drugs including IMIDs are used, e.g. LEN + dexamethasone [9], and long-lasting therapeutic treatments are required due to the difficulty of targeting the immune cells. However, these drugs can have serious side effects, among which neutropenia followed by thrombocytopenia are the most frequently reported ones [10]. Moreover, the reliance on stochastic events to deliver drugs to the tumour reduces their effectiveness. Some malignant cells have shown resistance to these drugs [3]. LEN has a short half-life (3–4 h). Besides, only a 20% of the oral dose is effective, and the rest is rapidly excreted via urine, which explains the need to adjust its dose, which has a higher risk of side effects in the renal system [11, 12]. In addition, the solubility of LEN in water and other solvents is limited. A controlled drug delivery to the tumour microenvironment could mitigate these issues. This action has pushed up the research of this drug to seek crystallographic forms of derivatives for increasing bioavailability, such as solvates, salts and two polymorphs of the crystal structures for the neutral molecule [13, 14]. The use of nanomaterials based on polymers and inorganic nanoparticles presents a novel approach for the development of drug delivery systems, including cancer therapy [15]. These nanomaterials have been explored for improving delivery, bioavailability and internalization of IMIDs into myeloma cells [16, 17]. Several nanocarriers have been searched for LEN, such as cyclodextrins [18], chitosan [19] and other polymers [20, 21].

Clay minerals are bio-sustainable minerals for environment-friendly applications owing to their high absorption capacity, great specific surface and small particle size, going to nanosized particles [22]. They are layered materials, 2-D materials, providing confined nanospaces with selective diffusion properties, which can alter the fluid dynamics of matter [23]. These properties have provided their applications as excipients for drug delivery nanomaterials [24, 25] with interesting pharmaceutical applications [26]. These materials are natural with low toxicity; however, the scaling-up to the industrial scale has been less developed than polymers [27]. The use of these minerals has not been applied to LEN yet. In this work, we propose their use for LEN-controlled delivery. The use of computational modelling of clay minerals at atomic and nanoscales is becoming an interesting methodology to explore and understand the crystal structure of these minerals [28] and the surface interactions of these minerals with drugs [29].

The main aims of this study are the following: (i) to find the intermolecular interactions being responsible for the energy and stability of the LEN crystal polymorphs and (ii) the adsorption on natural materials that can be used as excipients for increasing the LEN bioavailability, such as its intercalation into montmorillonite (MNT). Our calculations show that the intercalation of LEN into the confined interlayer space of MNT is possible considering the low solubility of this drug in aqueous media. We find that the use of clay minerals as an excipient for LEN formulations improves the bioavailability of this drug.

## Models

Two crystal structures of LEN were taken from experimental X-ray diffraction (XRD) data, where the racemic mixture is formed: polymorph LEN-1 (CCDC ref.755982) [15] and LEN-4 (CCDC ref. 1,487,162) [14].

To calculate the isolated LEN molecular structure, we set the LEN molecule in an 18 × 18 × 18 Å 3-D periodical box, in order to avoid intermolecular interactions with vicinal molecules.

An MNT crystal structure model was chosen with a chemical composition of the unit cell as Na(Si_7.83_Al_0.17_)(Al_3.17_Mg_0.83_)O_20_(OH)_4_ [30]. For the adsorption calculations, a 3 × 2 × 1 supercell was created obtaining a composition of Na_6_(Si_47_Al)(Al_19_Mg_5_)O_120_(OH)_24_. The octahedral Mg^2+^ cations were placed considering previous studies of cation ordering in smectites [29, 31]. Three water molecules per Na^+^ cation were placed in the interlayer space solvating the interlayer cations, resulting in Na_6_(Si_47_Al)(Al_19_Mg_5_)O_120_(OH)_24_(H_2_O)_18_.

Bulk water was modelled by an 18 × 18 × 18 3-D box, which was filled with 180 water molecules. Molecules were introduced with the Monte Carlo method up to reach a global density of 1 g/mL and optimized. In order to model LEN solved in water, one LEN molecule was set in the previous box of bulk water and optimized.

## Methodology

The INTERFACE force field (FF) [32] was used in our systems. This FF was mainly used in organic–inorganic systems with excellent results [33]. The COMPASS FF [34] also was used for comparison. We applied 3-D periodical boundary conditions (PBC), and the Forcite code was used for geometry optimizations within the Materials Studio package [35]. Non-bonding interactions, coulombic and (12–6) Lennard–Jones van der Waals potential were evaluated by using the Ewald summation method. Atomic charges based on electronegativities were calculated with the Qeq [36] and Gasteiger [37] methods. The harmonic vibrational frequencies were obtained with the Hessian matrix from finite atomic displacements. Molecular dynamics simulations were also performed at NVT ensemble with 1-fs steps during 5 ps.

In order to obtain alternative net atomic charges for the FF, ESP charges [38] associated to the electrostatic potential were obtained from quantum mechanical calculations based on density functional theory (DFT) by using the numerical atomic orbitals in Dmol^3^ code applying PBC [39]. The generalized gradient approximation (GGA) with the Perdew–Burke–Ernzerhof (PBE) [40] functional was used as a correlation exchange functional. Grimme G06 semi-empirical dispersion correction was used [41]. Pseudopotentials with semi-core correction (DSPP) were also used [36]. This approach has been applied previously successfully to similar systems [27]. The threshold for the convergence of energy at the self-consistent field (SCF) procedure was 10^−6^ Ha. Optimized geometries were characterized by harmonic vibrational frequencies, which were obtained from finite atomic displacements [36]. The results of the Hessian evaluation for the vibrational analysis of the thermodynamic properties can be computed as a function of temperature [36].

For comparative studies, plane-wave DFT calculations were also performed by using CASTEP code [42] with GGA and PBE approximations and norm-conserving pseudopotentials applying PBC. Grimme G06 semi-empirical dispersion correction was used [36]. This methodology was successfully applied previously in similar organic-clay-mineral composites [24]. The infrared spectroscopy frequencies were also studied in the CASTEP calculations and assigned to atomic vibration modes by DFPT [43] linear response of phonon calculations. From these phonon frequency calculations, thermodynamic properties, such as free energy, were analysed and the zero-point energy was considered.

## Results and discussion

### LEN molecular structure

**Fig. 2:**
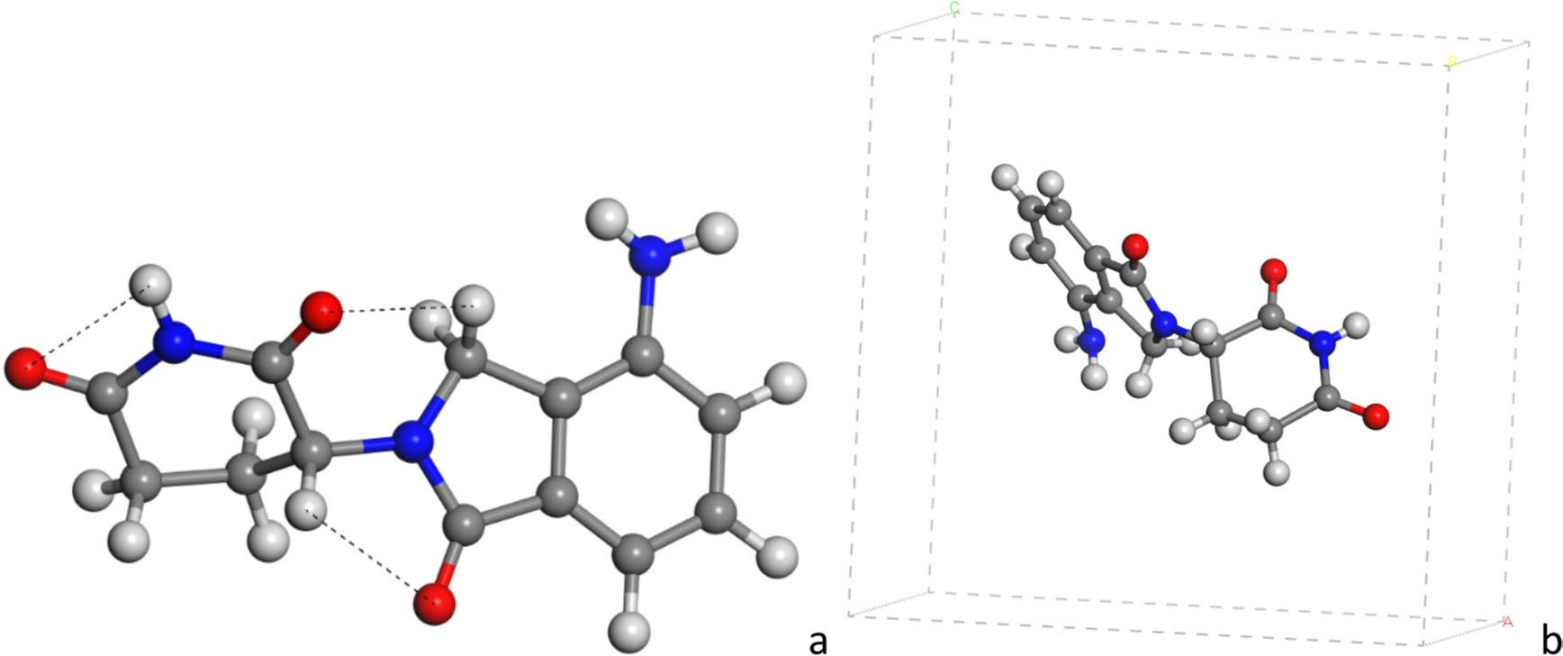
Optimized (CASTEP) molecular structure of LEN (**a**), embedded in a periodical box (**b**). The N, C, O and H atoms are represented in blue, grey, red and white colors, respectively. This colour representation is extended to all figures of this work. The main non-bonding interatomic interactions are highlighted with dash lines

The isolated molecule of LEN was calculated in the gas phase with the empty 3-D periodical box (18 × 18 × 18 Å) model applying PBC. The molecule was optimized with INTERFACE, Dmol^3^ and CASTEP methods yielding similar molecular structures (Fig. 2 and Fig. S1). However, small differences were observed in some non-bonding intramolecular interactions: (i) the carbonyl group of piperidine ring interacting with CH_2_ H atom of the other ring, *d*(CH…OC) = 2.54–2.47 Å; (ii) the carbonyl group of aromatic ring interacting with CH H atom of the piperidine ring, *d*(CH…OC) = 2.34–2.35 Å; and (iii) the aromatic ring forms a dihedral angle of (OC-C-N-CO) = 101.5º (CASTEP), 123.2º (Dmol^3^), 154.4º (INTERFACE) with respect to the piperidine ring, indicating that both rings are twisted and the carbonyl groups of aromatic and piperidine rings are not in the same side (Figs. 2 and 4a).

### LEN crystal polymorphs

Crystal structure calculations were carried out employing unit cells containing two LEN molecules for the LEN-1 polymorph and eight LEN molecules for the LEN-4 polymorph, arranged in the P1 space group. The optimization of the LEN crystal structures was performed with relaxation of atomic positions and lattice parameters, at a variable volume, using the INTERFACE and COMPASS FF with different net atomic charges and DFT calculations.

**Fig. 3:**
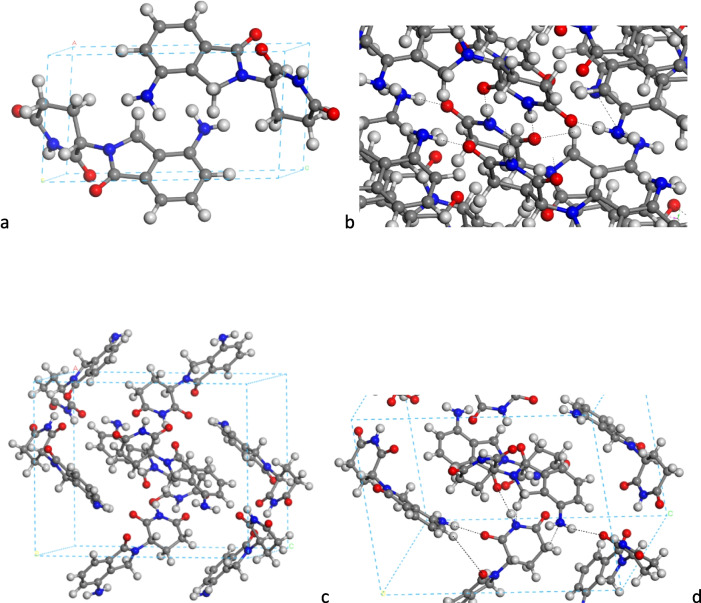
Optimized (INTERFACE) crystal structure of the LEN-1 (**a**, **b**) and LEN-4 (**c**, **d**) polymorphs. The main non-bonding interatomic interactions are highlighted with black dash lines

Table 1 presents a comparative analysis of the crystal lattice parameters for the optimized LEN-1 polymorph structure, considering various methods and relaxing all atom positions at variable volume, alongside experimental data. On the other hand, Table 2 provides similar insights for the LEN-4 polymorph. These tables offer valuable assessments of the precision exhibited by the different methods in reproducing experimental lattice parameters. Notably, the ESP atomic charges in the INTERFACE FF (INT_E) method successfully mirrored the experimental cell parameters for both polymorphs, thereby validating the suitability of this approach for the ensuing phases of the present work (Fig. 3). Besides, these values are similar to those obtained with DFT.

**Table 1:** Experimental and fully optimized values of lattice cell parameters (distances in Å and angles in degrees) for polymorph LEN-1

Param	EXP^a^	DFT^b^	INTERF^b^	INT_Q^b^	INT_G^b^	INT_E^b^	CF^c^	CF_Q^c^	CF_G^c^	CF_E^c^
*a*	6.00	5.98	6.00	6.16	6.07	5.88	5.29	6.27	6.25	5.92
*b*	8.92	8.60	8.96	8.72	8.97	9.01	10.32	8.58	8.75	8.87
*c*	11.58	11.67	11.40	11.34	11.64	11.70	11.19	11.11	11.29	11.56
ME^d^		0.08	0.05	0.09	− 0.06	− 0.03	− 0.10	0.18	0.07	0.05
MAE^d^		0.14	0.07	0.20	0.06	0.11	0.83	0.36	0.24	0.05
*α*	75.7	76.7	77.3	77.3	74.7	76.3	80.3	78.7	76.5	76.8
*β*	84.7	84.5	86.0	84.7	82.6	84.0	89.8	83.6	82.6	83.5
*γ*	86.0	87.6	87.2	84.7	85.5	86.2	93.5	83.6	84.8	87.0
ME^d^		− 0.8	− 1.37	− 0.1	1.2	− 0.03	− 5.73	0.17	0.83	− 0.3
MAE		0.93	1.37	0.97	1.2	0.50	5.73	2.17	1.37	1.1

^a^From ref. [15]. ^b^*DFT*, at DFT level with CASTEP; INTERF, INTERFACE FF with their own atomic charges; *INT_Q*, INTERFACE FF with atomic charges calculated with the Qeq method; INT_G, INTERFACE FF with atomic charges calculated with the Gasteiger method; INT_E, INTERFACE FF with ESP atomic charges calculated with Dmol^3^ DFT. ^c^CF, compass FF with their own atomic charges; CF_Q, compass FF with atomic charges calculated with the Qeq method; CF_G, compass FF with atomic charges calculated with the Gasteiger method; CF_E, COMPASS FF with ESP atomic charges calculated with Dmol^3^ DFT. ^d^ME, mean relative difference with experimental values; MAE, mean absolute difference with experimental values

**Table 2:** Comparison of experimental cell parameters (in Å) of the LEN-4 polymorph with the fully optimized values

Param.^a^	EXP^b^	DFT^c^	INTERF^c^	INT_Q^c^	INT_G^c^	INT_E^c^	CF^d^	CF_Q^d^	CF_G^d^	CF_E^d^
*a*	12.95	12.92	13.29	11.32	12.29	13.11	13.60	11.16	12.96	13.18
*b*	11.12	11.11	11.05	11.39	11.29	10.99	10.69	11.31	11.03	10.72
*c*	16.73	16.63	16.36	18.96	18.24	16.63	15.85	18.75	16.99	16.35
ME^e^		0.05	0.03	− 0.29	− 0.34	0.02	0.22	− 0.14	− 0.06	0.18
MAE^e^		0.05	0.26	1.38	0.78	0.13	0.65	1.33	0.12	0.34

^a^Cell parameters, in all cases, the angles *α*, *β* and *γ* are 90º. ^b^From ref. [14]. ^c^*DFT*, at DFT level with Castep; INTERF, INTERFACE FF with their own atomic charges; *INT_Q*, INTERFACE FF with atomic charges calculated with the Qeq method; INT_G, INTERFACE FF with atomic charges calculated with the Gasteiger method; INT_E, INTERFACE FF with ESP atomic charges calculated with Dmol^3^ DFT. ^d^CF, COMPASS FF with their own atomic charges; CF_Q, COMPASS FF with atomic charges calculated with the Qeq method; CF_G, COMPASS FF with atomic charges calculated with the Gasteiger method; CF_E, COMPASS FF with ESP atomic charges calculated with Dmol^3^ DFT. ^e^ME, mean difference with experimental values; MAE, mean absolute difference with experimental values

The dihedral angle between the aromatic rings (OC-C-N-CO) changes significantly in the crystal form LEN-1, being (OC-C-N-CO) = 90.2º (INTERFACE), 83.4º (CASTEP) compared with the isolated molecule. In this LEN-1 polymorph, the main intermolecular interactions are hydrogen bonds (Fig. 3a, b). Amine H atoms interact with amine N atoms with *d*(N…H) of 2.387 Å (INTERFACE). Furthermore, interactions also occur between amino H atoms and the O atoms of the piperidine carbonyl group in γ position to the aromatic ring, *d*(O…H) = 1.917 Å (INTERFACE). Additionally, the carbonyl group of the piperidine ring in α position to the aromatic ring forms a hydrogen bond with the amide H atom of the piperidine ring, *d*(NH…OC) = 1.796 Å (INTERFACE). The heterocyclic carbonyl group interacts also with the vicinal piperidine CH_2_ groups, *d*(CH…OC) = 2.472–2.605 Å (INTERFACE). The aromatic rings are co-planar but shifted at 3.46 Å (INTERFACE). An intermolecular motif is observed between the amide NH and carbonyl O atoms NH…OC-NH…OC of two vicinal molecules, remarking the high tautomerism of this NH H atom. This strong interaction is responsible for the change in the (OC-C-N-CO) dihedral angle. The other carbonyl group interacts with the amine group of a third molecule. These geometrical features are similar to the experimental ones.

In the LEN-4 polymorph, the aromatic rings are quasi-perpendicular to each other (Fig. 3c, d) and not in parallel planes as in LEN-1. The dihedral angle between aromatic and piperidine rings is similar, (OC-C-N-CO) = 85.6º (INTERFACE). The amide H atom forms strong hydrogen bonds with the heterocycle carbonyl O atom, *d*(CO…HN) = 1.785 Å (INTERFACE). The amine H atoms form also hydrogen bonds with piperidine carbonyl O atoms, *d*(CO…HN) = 1.877 Å (INTERFACE).

Comparing the energies of the isolated molecule and the two crystal forms, we calculated the packing energy (INTERFACE), being − 44.70 and − 44.40 kcal/mol per LEN molecule for the polymorphs LEN-1 and LEN-4, respectively. Similar values were obtained with DFT calculations, − 44.62 (CASTEP) (− 48.73 Dmol^3^) and − 44.24 (CASTEP) (− 48.65 Dmol^3^) kcal/mol per LEN molecule for LEN-1 and LEN-4, respectively. Both crystal forms behave energetically with a similar packing energy. However, the packing free energy calculated by means of phonon calculations with CASTEP is + 1.06 kcal/mol for LEN-1 and − 0.06 kcal/mol for LEN-4. Nevertheless, this small energy difference can explain the formation of concomitant polymorphs of both forms during crystallization, and the driving forces to obtain one polymorph will come mainly from the experimental conditions [18].

**Fig. 4:**
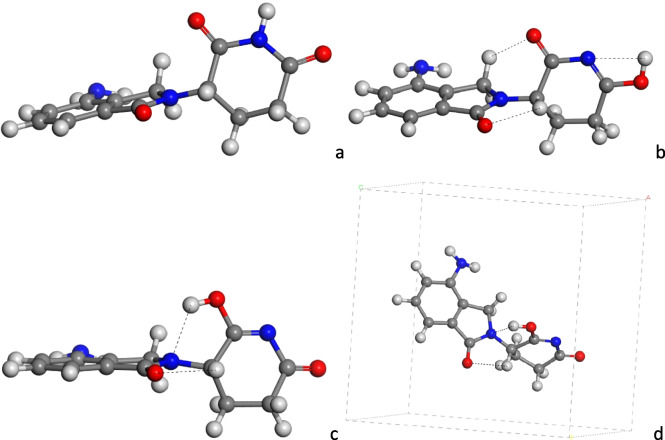
Molecular structure of tautomers of LEN optimized by DFT, amide (**a**), iminol-tauA (**b**) and iminol-tauB (**c**) and embedded in a 3-D periodical cell (**d**). The main non-bonding interatomic interactions are highlighted with black dash lines

Considering the intermolecular interactions observed in these crystals, possible amide-iminol tautomers were explored as isolated molecules at DFT level (Dmol^3^) applying PBC: the initial dioxopiperidine (amide form) (Fig. 4a), the inimol-tauA (Fig. 4b) and the iminol-tauB (Fig. 4c, d). The piperidine ring is twisted with respect to the aromatic ring in the three tautomers, forming dihedral angles between the carbonyl groups (OC-C-N-CO) of 115.4º, 129.9º and 120.8º for amide, iminol-tauA and iminol-tauB, respectively. These structures are stabilized by intramolecular interactions, *d*(CO…HC) = 2.33–2.49 Å (amide), 2.35 Å (iminol-tauA), 2.35–2.40 Å (iminol-tauB), and *d*(COH…NC) = 2.102 Å (iminol-tauA), and 2.24 Å (iminol-tauB).

The amide form, dioxopiperidine, is 17.17 and 21.7 kcal/mol more stable than both tautomers iminol-tauA and iminol-tauB, respectively. However, considering the differences in free energy calculated with Dmol^3^ (Δ*G*) at 298 K, the tautomer iminol-tauB is 0.48 and 1.26 kcal/mol more stable than the dioxopiperidine and iminol-tauA respectively. This can explain the tendency to form intermolecular interactions of this NH group in the crystal structures.

### Hydration energy

**Fig. 5:**
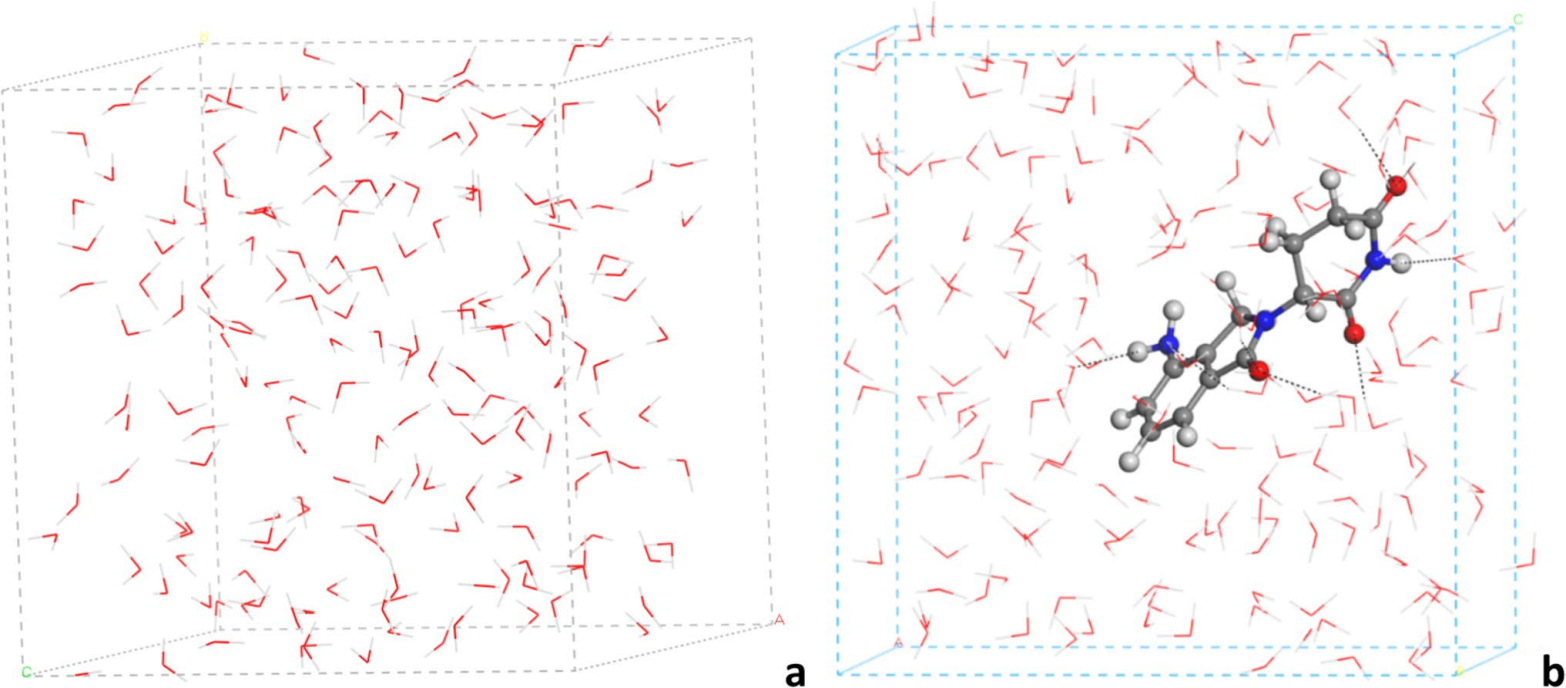
Optimized structure of 3-D periodical cells of water (**a**) and LEN molecule hydrated (**b**)

Bulk water model was performed with the 18 × 18 × 18 Å 3-D periodical cell filled with 180 water molecules at 298 K, and the system was optimized with the INTERFACE FF (Fig. 5a). In the hydrated LEN-water model, the LEN molecule is mixed with the previous bulk water 3-D periodic cell model and optimized with the INTERFACE FF (Fig. 5b). The hydration energy was calculated by difference, such as:

1 $${E}_{\text{hydr}}={E}_{\text{LEN}-\text{water}}-{E}_{\text{LEN}}-{E}_{\text{water}}$$

being − 99.05 kcal/mol. Considering that the dissolution of a LEN solid in crystalline form (LENcryst) will be:

2 $${E}_{\text{dissol}}={n}^{*}{E}_{\text{LEN}-\text{water}}-{E}_{\text{LENcryst}}-{n}^{*}{E}_{\text{water}}$$

being *n* the number of LEN molecules per unit cell in each crystal. Hence, the dissolution energy will be − 54.36 and − 34.97 kcal/mol per LEN molecule for LEN-1 and LEN-4, respectively. These values are similar to the packing energy of these polymorphs.

The dihedral angle between rings in the hydrated state is (OC-C-N-CO) = 118.4º. Some water molecules interact with the main functional groups of LEN. The amino H atoms form hydrogen bonds with water O atoms, *d*(NH…OH) = 2.107 Å, and water H atoms are interacting with the amino N atom, *d*(OH…N) = 2.223 Å. The piperidine NH group forms hydrogen bonds with water O atoms, *d*(NH…OH) = 2.113 Å. The carbonyl O atoms also form hydrogen bonds with water H atoms, *d*(C = O…HO) = 1.827–1.907 Å (heterocyclic C = O), 1.899 and 1.963–2.065 Å (piperidinic C = O).

### Intercalation of LEN into montmorillonite

**Fig. 6:**
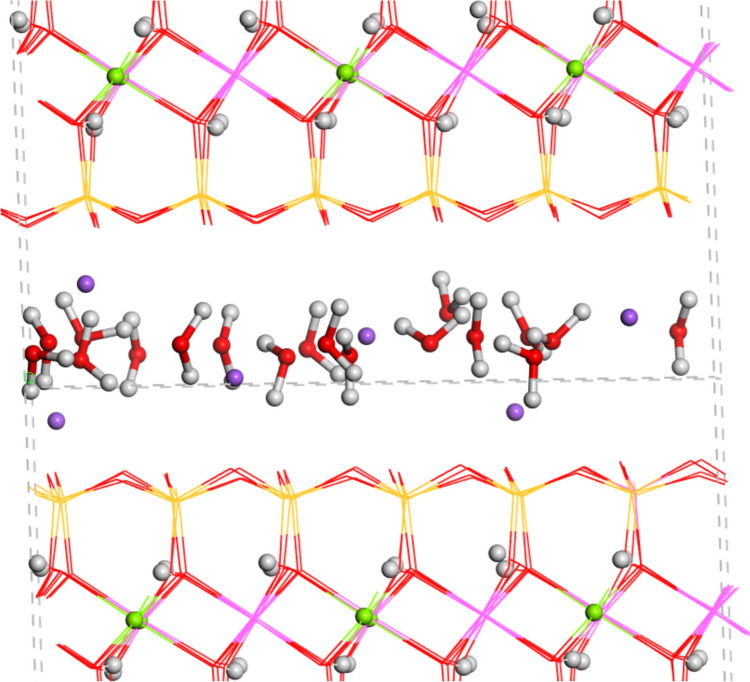
Optimized crystal structure of MNT. The H, Mg, Na and water atoms are highlighted as balls. The Si, Al, Mg and Na atoms are represented in yellow, pink, green and fuchsia colours, respectively. This colour representation style is extended to all figures of this work

The crystal structure of MNT was fully optimized (atomic positions and crystal cell parameters) with PBC yielding an interlayer space of 11.6 Å according to previous studies of MNT with interlayer hydrated Na^+^ cations [44]. In the interlayer space, the water O atoms are coordinating the Na^+^ cations and the water H atoms are oriented to the basal O atoms of the interlayer mineral surface (Fig. 6).

**Fig. 7:**
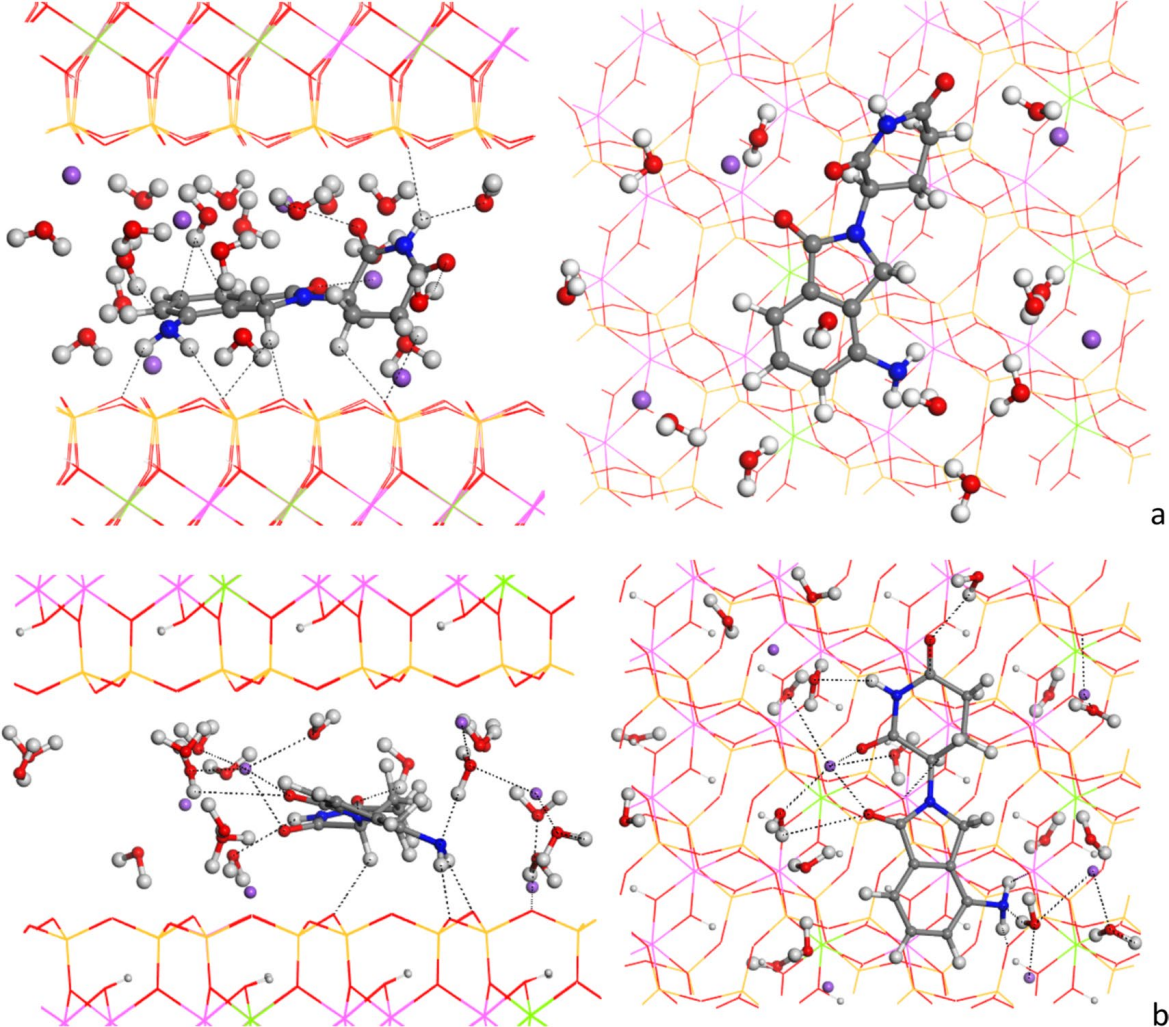
Adsorption complex MNT-LEN optimized with INTERFACE with one LEN molecule per 3 × 2 × 1 supercell of MNT, before (**a** views from (010) and (001) planes) and after (**b** views from (100) and (001) planes) molecular dynamics simulations. The main non-bonding interatomic interactions are highlighted with black dash lines

One LEN molecule was placed in the centre of the interlayer space of MNT, ensuring adequate room for the subsequent intercalation process. Afterward, the composite was fully optimized with INTERFACE FF with PBC (MNT-LEN). After optimization, the interlayer space notably expanded after intercalation, transitioning from its initial measurement of *d*(001) at 11.6 Å in Na^+^ MNT to 14.7 Å in MNT-LEN. The dihedral angle between the aromatic and piperidine rings of the intercalated LEN molecule was maintained being close to that of crystal structure, (OC-C-N-CO) = 75.2º (INTERFACE). Throughout the optimization, the LEN molecule remained in the centre of the interlayer space, demonstrating clear-cut interactions with both the tetrahedral basal O (O_b_) atoms on the mineral surface and the surrounding water molecules and Na^+^ cations located in the interlayer space (Fig. 7). Several H atoms in the LEN molecule interact with the nearby O_b_ within the range of 1.95–2.64 Å. Hydrogen bonds also formed between the amide and carbonyl groups of the piperidine ring in LEN and adjacent water molecules, at interaction distances of approximately *d*(NH…OH_2_) = 2.11 Å and *d*(CO…H_2_O) = 1.88–2.22 Å. Concerning the aromatic ring, the amine N atom established a hydrogen bond with a water H atom, *d*(H_2_N···HOH) = 1.96–2.05 Å. The amine H atoms also have interactions with the O_b_ atoms of mineral surface, *d*(NH_2_…OSi) = 2.46–2.64 Å. The Na^+^ cations in the closest proximity to LEN exhibit coordination with the carbonyl groups of the aromatic and piperidine rings in LEN, with distances of *d*(O…Na) measuring 2.30 and 2.46 Å, respectively (Fig. 7a).

In order to explore different adsorption positions of LEN into the 3 × 2 × 1 clay interlayer space model, molecular dynamics simulations were performed within INTERFACE FF and the NVT ensemble with steps of 1 fs during 5 ps (5000 steps) (see movie S1 in Supplementary material). From the sampling of this simulation, we explored the energy of the 51 snapshots selected choosing the lowest energy sample. After dynamics, a complete optimization of the system was performed, obtaining a more stable complex than above with lower energy than the previous system with *d*(001) = 13.9 Å. In this complex, the LEN molecule piperidine and aromatic ring are approximately planar, where both carbonyl groups are oriented to the same side with (OC-C-N-CO) = 2º (Fig. 7b).

The interlayer space obtained for the MNT-LEN complex can be a good target to follow during the intercalation experiments by XRD analysis where the MNT intercalated with LEN should reach a *d*(001) value close to or higher than 13.9 Å.

From this model, the intercalation energy of one LEN molecule in the interlayer space of MNT can be determined through the following equation:

3 $${E}_{\text{int}}={E}_{\text{MNT}-\text{LEN}}-{\text{E}}_{\text{MNT}}-{\text{E}}_{\text{LEN}}$$

being for a single LEN molecule into the interlayer space of MNT + 0.39 kcal/mol, which indicates that LEN can be intercalated into MNT kinetically. Considering that the hydration energy (Eq. (1)) and dissolution energy (Eq. (2)) of LEN are more exothermic processes than intercalation in MNT, the intercalation process should be easier by using non-aqueous solvents. This result can be useful for future experimental studies.

**Fig. 8:**
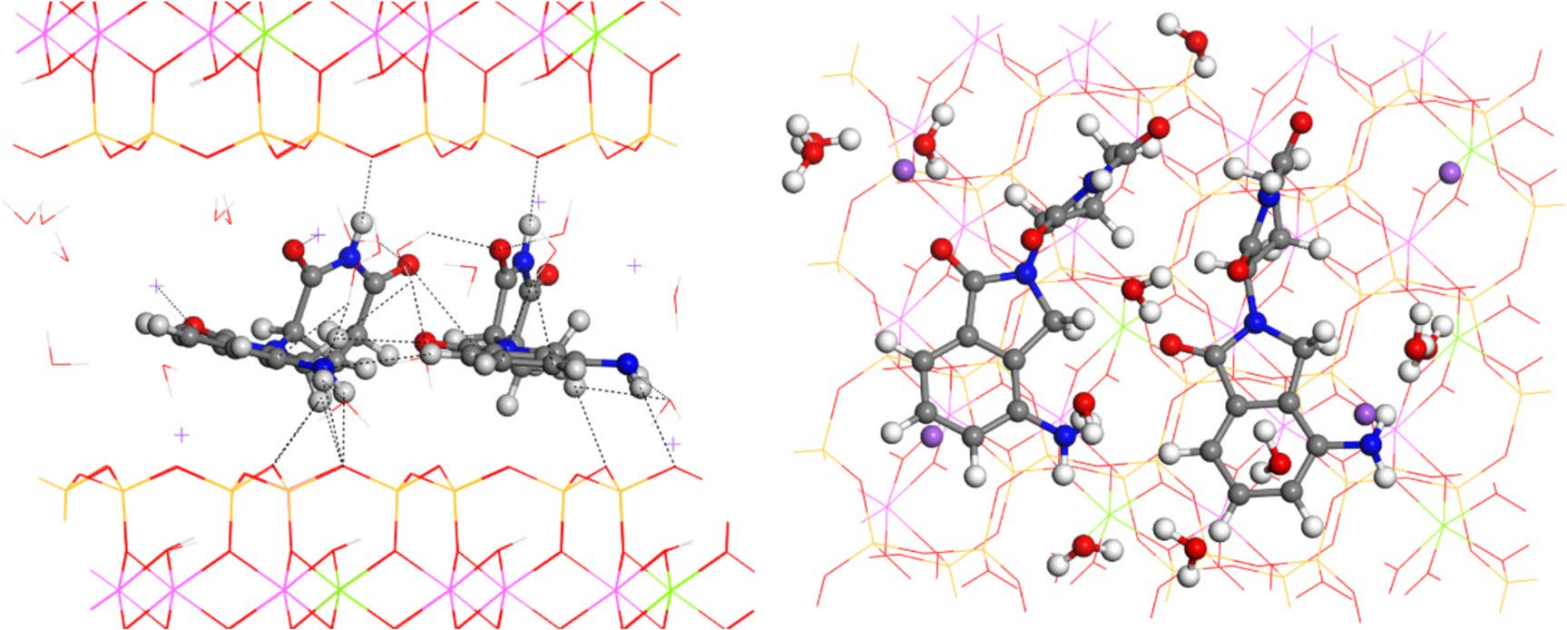
Adsorption complex MNT-2LEN optimized with INTERFACE with two LEN molecules per 3 × 2 × 1 supercell, views from (010) and (001) planes. The main non-bonding interatomic interactions are highlighted with black dash lines

Two LEN molecules per 3 × 2 × 1 supercell (MNT-2LEN) were intercalated in the interlayer space and subsequently optimized with INTERFACE FF as well. This adsorption complex with two LEN molecules displayed similar interactions to those previously described within the MNT-LEN complex, with the additional feature of manifesting intermolecular interactions between LEN molecules (Fig. 8). The interlayer space shows a notable expansion to *d*(001) = 15.1 Å. This result can be a good guide for experimental intercalation processes of LEN in MNT, where different interlayer spacing will indicate different proportions of LEN in MNT. The two molecules exhibited similar dihedral angles (OC-C-N-CO) between their aromatic and piperidine rings, 83.7º and 94.2º. As a consequence of these dihedral angles, the interlayer space is larger than MNT-LEN. The carbonyl O atom of the piperidine group interacts with water H atoms, *d*(CO…OH_2_) = 1.96–2.06 Å. Additionally, the carbonyl O atom of the aromatic ring formed a hydrogen bond with the H atom of CH_2_ in the adjacent LEN molecule, with a distance of *d*(CO…CH_2_) = 2.44 Å. Carbonyl groups interact with Na^+^ cations at distances of *d*(CO…Na) = 2.36–2.44 Å. Both molecules establish hydrogen bonds with O_b_ of the mineral surface with 2.09–2.55 Å distances. Furthermore, both molecules formed several hydrogen bonds with water molecules through the amine groups, *d*(NH_2_…OH_2_) = 1.88–2.31 Å, and carbonyl groups, *d*(CO…OH_2_) = 1.74–2.06 Å. Additional intermolecular interactions involved the piperidine rings of both molecules, where the carbonyl O atom interacted with the H atom of the CH_2_ group, *d*(CO…CH_2_) = 2.84 Å. In the same way, we find a *d*(CO…CH_2_) = 2.44 Å interaction and some other interactions involving the N atom of the amine group and the H atom of the CH group from the vicinal molecule, at *d*(NH_2_…CH) = 2.96 Å.

The adsorption energy of the two LEN molecules in the interlayer space of MNT can be determined through the following equation:

4 $${E}_{\text{int}}={E}_{\text{MNT}-2\text{LEN}}-{E}_{\text{MNT}}-2*{E}_{\text{LEN}}$$

The intercalation energy of two LEN molecules per 3 × 2 × 1 supercell of MNT was − 5.21 kcal/mol, being energetically favourable, and − 2.60 kcal/mol per LEN molecule. So, the presence of the second LEN molecule increases the lipophilicity of the interlayer space and the adsorption capacity without water. So, our calculations recommend an intercalation process by using non-aqueous media.

### Release of LEN

**Fig. 9:**
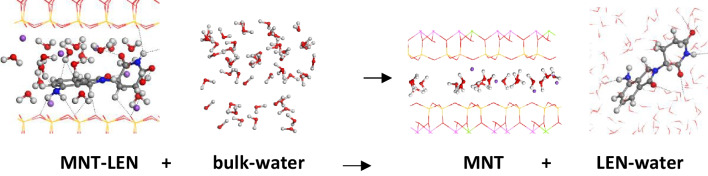
Delivery process of LEN from the MNT-LEN hybrid complex with INTERFACE calculations

After the intercalation of LEN into MNT, LEN can be delivered to the aqueous media. Indeed, we can simulate the transfer of LEN intercalated in the interlayer space of a clay mineral to an aqueous media. This process is accomplished with INTERFACE FF. This system was simulated by a periodical box with one LEN molecule in a box of 180 water molecules, and the water media was modelized with an equal box with only 180 water molecules, with the following process (Fig. 9):

5 $$\text{MNT}-\text{LEN}+\text{bulk}-\text{water}\leftrightarrow \text{MNT}+{\text{LEN}-\text{water}}$$

The delivery energy can be determined by using the equation:

6 $${E}_{\text{deliver}}={E}_{\text{MNT}}+{E}_{\text{LEN}-\text{water}}-{E}_{\text{MNT}-\text{LEN}}-{E}_{\text{water}}$$

The delivery energy for one LEN molecule from a 3 × 2 × 1 supercell of MNT was − 99.45 kcal/mol, being highly energetically favourable and of the same order as the hydration energy (Eq. (1)). In the case of the hybrid composite with two LEN molecules per 3 × 2 × 1 supercell of MNT, the delivery energy is − 192.90 kcal/mol, − 96.45 kcal/mol per LEN molecule. Besides, this energy is approximately twice the packing energy of both LEN polymorphs and the dissolution energy of LEN (Eq. (2)), indicating that the LEN delivery from the MNT-LEN complex is much more exothermic than directly from the crystal forms of LEN. Therefore, the energetic expenses to intercalate LEN from a non-aqueous solvent into MNT are energetically favourable, and the desorption from MNT to an aqueous media is much more energetically favourable. Besides, the unpacking energy is a process needing energy to put LEN in molecules. Therefore, the process for delivering LEN from the MNT-LEN complex into water is more available than that coming from crystal to free molecules and dissolve it in water and more bioavailable in consequence. Considering these calculations, methodology, items and discussions, it is possible to suggest a pharmacological preparation of MNT-LEN to be more bioavailable in a water medium than traditional pharmacological pill preparations.

### Spectroscopic IR properties

In addition to the XRD analysis, the infrared (IR) spectroscopy is a useful experimental technique for identifying molecules and controlling the intercalation process of organics into clay minerals. This technique provides frequency values produced by atomic vibrations showing information on the structure and environment of functional group vibrations at the atomic scale. Hence, this technique can be directly connected with calculations at the atomic level, which can be used for interpreting the bands observed in experimental spectra [25]. The frequencies of the main vibration modes were calculated in the optimized structures of isolated molecule, crystal polymorphs and intercalated composite. These frequencies have been calculated with structures optimized at Dmol^3^ (Table 3), CASTEP and INTERFACE FF (see Supplementary Material). These values were compared with experimental ones (Table 3). A good agreement is found between the frequencies of optimized LEN crystal structures and experimental values which were analysed also at solid state in their LEN crystal forms.

**Table 3:** IR frequencies (in cm^−1^) calculated from the optimized (Dmol^3^) structures comparing with experimental values

Mode^*a*^	Exp	LEN	LEN-1	LEN-4	MNT-LEN
ν(OH)					3778–3504, 3483–3411, 3282, 3198–3182
ν(NH_2_)_*as*_	3475–3310^b^, 3407^ k^, 3455^ m^	3610	3498–3495	3566–3560	3489
ν(NH_2_)_*s*_	3342^ k^, 3362^ m^	3497	3405–3399	3420–3416	3415
ν(NH)	3250–3140^b^, 3080^ k^	3475	3059	3126–3112	3060
ν(CH)_arom_	3080–3050^b,c,d^, 3185–3148^ k^, 3153^ m^	3184^c^, 3169^d^, 3159^e^,	3182^e^, 3174^d^, 3136^c^	3199^c^, 3181^d^, 3150^e^	3252^e^, 3192_s_^c,d^, 3180_as_^c,d^
ν(CH_2_)_*as*_	2975–2840^b^, 3080^ k^, 3057^ m^	3082–3078, 3013^ g^,	3097–3091, 3008^ g^,	3102^f^, 3018^f^, 3094^ h^, 3035^ g^	3121^ h^, 3115^f^ax, 3061^ g^, 3035^f^, 3021^h^ax
ν(CH_2_)_*s*_	2964^ k^	3025^f^, 2990^ h^, 2945^ g^	3046^f^, 2993^ h^, 2969^ g^	2992^ g^, 2959^ h^	2999^ g^,
ν(C–H)	2964–2879^ k^, 2841^ m^	2957	2967–2964	2979	2959
ν(C = O)	1740–1620^b^, 1700–1640^ k^, 1702–1675^ m^	1751^i^, 1740^f^, 1731^ g^	1702_s_^j^, 1682_as_^j^, 1693^i^, 1669^f^, 1642^ g^	1709–1696. 1691^f^, 1689^f,h^, 1682^ g^, 1671–1663^ g^	1709^f^, 1689^ g^, 1673^i^
δ(NH_2_)_*s*_	1630–1603^ k^, 1633–1607^ m^	1634–1610	1639–1622	1635–1598	1635
ν(C = C), ring	1610–1400^b^	1607	1591–1581	1588–1586	1622–1594, 1400
δ(CH)	1486^ k^, 1493^ m^	1488^c–d^	1477–1474^c–d^	1496–1493^c–d^	1478
δ(CH_2_)_*s*_	1460^ k^, 1445^ m^	1469^ g^, 1452–1450^f^, 1430^ h^	1460–1457^f^, 1454^ g^, 1405^ h^	1467–1464^f^, 1458–1454^ g^, 1445^ h^	1471^ g^, 1459^f^, 1442^ h^
δ(NH)	1460^ k^, 1675–1445^ m^	1409	1479	1688–1686, 1680–1671, 1434	1461
δ(CH)	1341–1241^ k^, 1350–1169^ m^	1396^ g^, 1382–970	1374, 1284	1351	1380–1203
γ(NH)			956	946–943	922
γ(CH)	880^ k^, 879–746^ m^	949–880	930–845	794	956
γ(NH_2_)_*s*_	739^ k^		746		

^a^Notation: *s*, symmetrical; *as*, asymmetrical; *ν*, stretching vibration; *δ*, bending in-plane vibration; *γ*, bending out-of-plane vibration; ax, axial. ^b^Interpreted by Chennuru et al. [14]. ^c^Aromatic in meta position with respect to the amine group. ^d^Aromatic in ortho position with respect to amine. ^e^Aromatic in para position with respect to amine. ^f^Piperidine ring in position α with the aromatic moiety. ^g^In the heterocyclic ring. ^h^Piperidine ring in position β with the aromatic moiety. ^i^Piperidine ring in position γ with the aromatic moiety. ^j^O = C–N–C = O. ^k^Extracted from the spectra of LEN-1 [15]. ^m^Extracted from the spectra of LEN-4 [14]

**Fig. 10:**
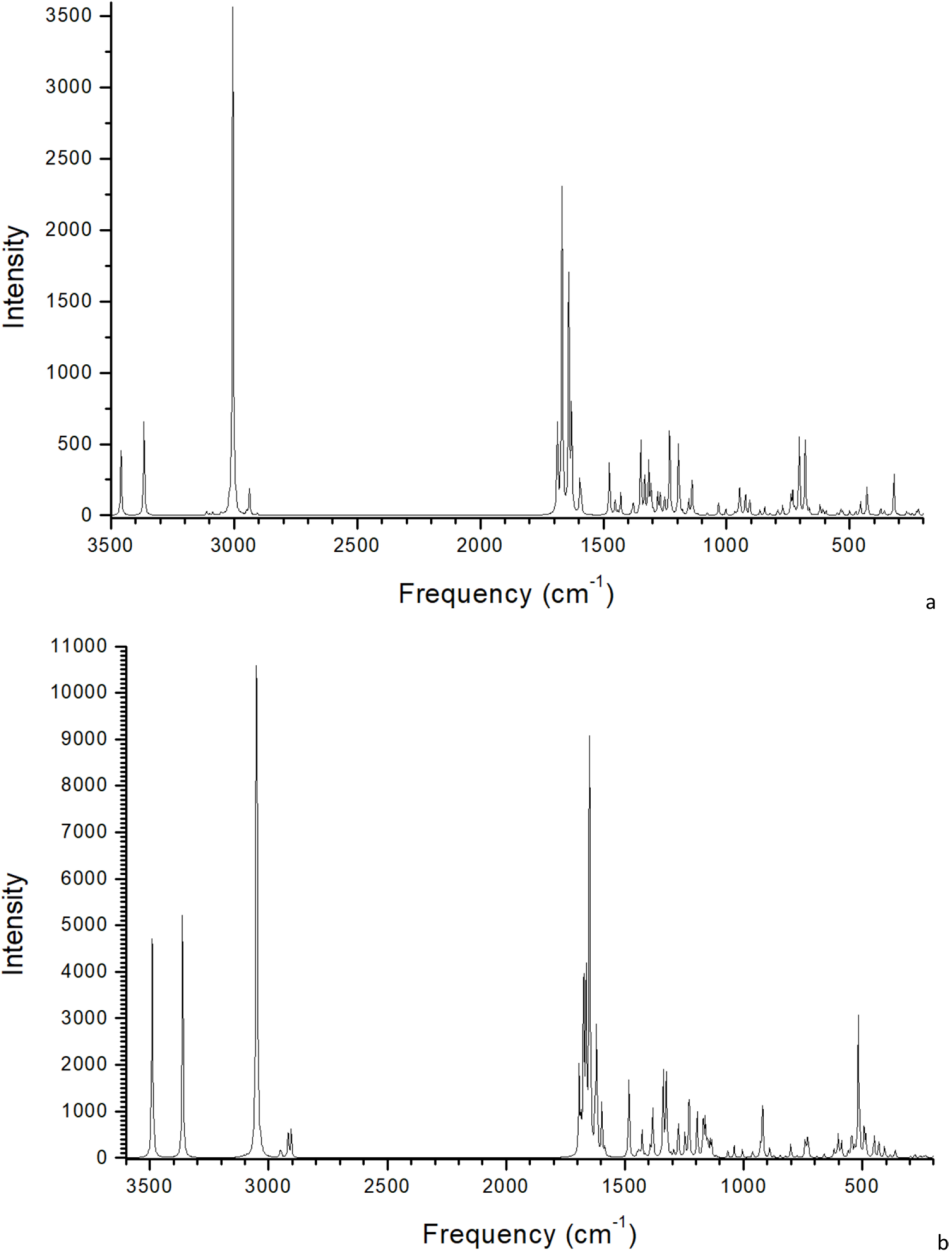
Calculated IR spectra of the optimized LEN-1 (**a**) and LEN-4 (**b**) polymorphs calculated with Dmol^3^

The bands at 3778–3504 cm^−1^ in MNT-LEN can be assigned to the stretching ν(OH) vibration mode of the OH groups of MNT depending on the position of the cation substitution in the octahedral and tetrahedral sheets, according to previous works [32]. Bands at 3483–3411, 3282 and 3198–3182 cm^−1^ correspond to the ν(OH) of the water molecules that have different interactions with interlayer cations or the O atoms of the internal mineral surface. A stronger interaction yields a longer bond length and its stretching vibration mode has a lower frequency. In this region, some bands can be overlapped, such as at 3489 and 3415 cm^−1^, which are assigned to the ν(NH) mode in MNT-LEN. This band appears at a lower frequency than in the LEN molecule. Similar behaviour is detected in the LEN crystal forms. Then, a drastic shift to lower frequencies is observed in ν(NH) of N–H bonds from the isolated molecule to the crystal lattice of LEN polymorphs and in the MNT-LEN complex, indicating that these groups are responsible for the packing interactions in both polymorphs and in the interactions of LEN with the interlayer clay surfaces. This effect is due to the hydrogen bond formation and is observed in the amine and NH groups, being stronger in the amide NH group. This frequency shift is higher in LEN-1 than in LEN-4 (Fig. 10). On the contrary, a high-frequency shift is shown in the ν(CH) bands produced by electrostatic interactions during the vibration mode in the LEN crystal polymorphs and MNT-LEN complex (Table 3). The ν(C = O) bands show a low-frequency shift in the formation of polymorph crystals and in the intercalation of LEN in the clay mineral, due to the hydrogen bonds with these carbonyl groups. Therefore, the observation of frequency shifts during the experimental intercalation process can indicate that LEN is intercalated into the MNT interlayer space.

## Conclusions

Our calculations are the first computational study at the atomic scale of the periodic crystal forms of the polymorphs for the LEN anticancer drug, highly demanded in the pharmacy market. This study has compared different FF and DFT calculations for the crystal structure of LEN, indicating that INTERFACE FF is suitable for determining the structure and energy of periodical systems, yielding similar results to those from high-level DFT calculations.

Our calculations provide useful information for experimental aspects of the preparation of hybrid MNT-LEN composites, for the delivery of LEN from the hybrid composite, and the process could be monitored by IR spectroscopy and X-ray diffraction techniques. In the preparation process, our results recommend the use of non-aqueous media for the intercalation of LEN into MNT. On the other hand, the delivery of LEN from the hybrid composite can be used in aqueous media. Both steps are energetically favourable. This process can be controlled by X-ray diffraction (XRD) and IR spectroscopy. The intercalation process can be confirmed by observing the expansion of the *d*(001) spacing value from 11.6 of MNT to 13.9–14.9 Å in the MNT-LEN complex and 15.1 Å in the MNT-2LEN complex.

The intermolecular interactions in the crystal structures of both LEN polymorphs, compared with the isolated molecule, produce low-frequency shifts of several bands in the IR spectra, whose functional groups are forming hydrogen bonds. Similar interactions are observed in the LEN intercalated in MNT, and so, similar frequency shifts are observed, being higher than the LEN-4 polymorph. Hence, the observation of low-frequency shifts in the IR spectra during the intercalation process can be an experimental indication that the LEN molecule has been intercalated into MNT interlayer space.

The LEN molecule adopts a quasi-planar configuration into the confined interlayer space of MNT forming a mono-layer of drug, even with two LEN molecules per 3 × 2 × 1 supercell of clay mineral (around 10% w/w), although configuration leaves the quasi-planar configuration.

Intercalation of LEN into the interlayer space of MNT is energetically favourable, suggesting LEN should be dissolved in a non-aqueous solvent. Besides, delivery of LEN from the interlayer space of MNT to water solvent is a recommended process, whereupon a pharmacological preparation of LEN in MNT will be much more bioavailable than from the LEN crystal form.

All in all, this work indicates that the anticancer drug lenalidomide can be intercalated into MNT and this composite can be a good delivery system for increasing the bioavailability of LEN in the anticancer treatments.

## Supplementary Information

Below is the link to the electronic supplementary material.

Supplementary file1 (MP4 3313 KB)

Supplementary file2 (DOCX 471 KB)

## Data Availability

Data will be provided by request to the corresponding authors.
