# Supplementary material for: Intercalation of the anticancer drug lenalidomide into montmorillonite for bioavailability improvement: a computational study
Source: J Mol Model. 2024 Dec 4;31(1):5. doi: 10.1007/s00894-024-06210-w (PMC11618151; doi:10.1007/s00894-024-06210-w)
Supplement: Supplementary file 2 — Supplementary file2 (DOCX 471 KB) [file 894_2024_6210_MOESM2_ESM.docx]

**SUPPLEMENTARY INFORMATION**

**Intercalation of the anticancer drug lenalidomide into montmorillonite for bioavailability control. A computational study.**

Yumeida V. Meruvia-Rojas*^a,b^*, Esther Molina-Montes*^b,c^* Alfonso Hernández-Laguna*^a^*, C. Ignacio Sainz-Díaz*^a^*

*^a^* Instituto Andaluz de Ciencias de la Tierra, CSIC-UGR, Av. de las Palmeras, 4, 18100-Armilla, Granada, Spain.*^b^* Pharmacy Faculty, University of Granada, Granada, Spain.

*^C^* Instituto de Investigación Biosanitaria ibs. GRANADA, Granada, Spain.


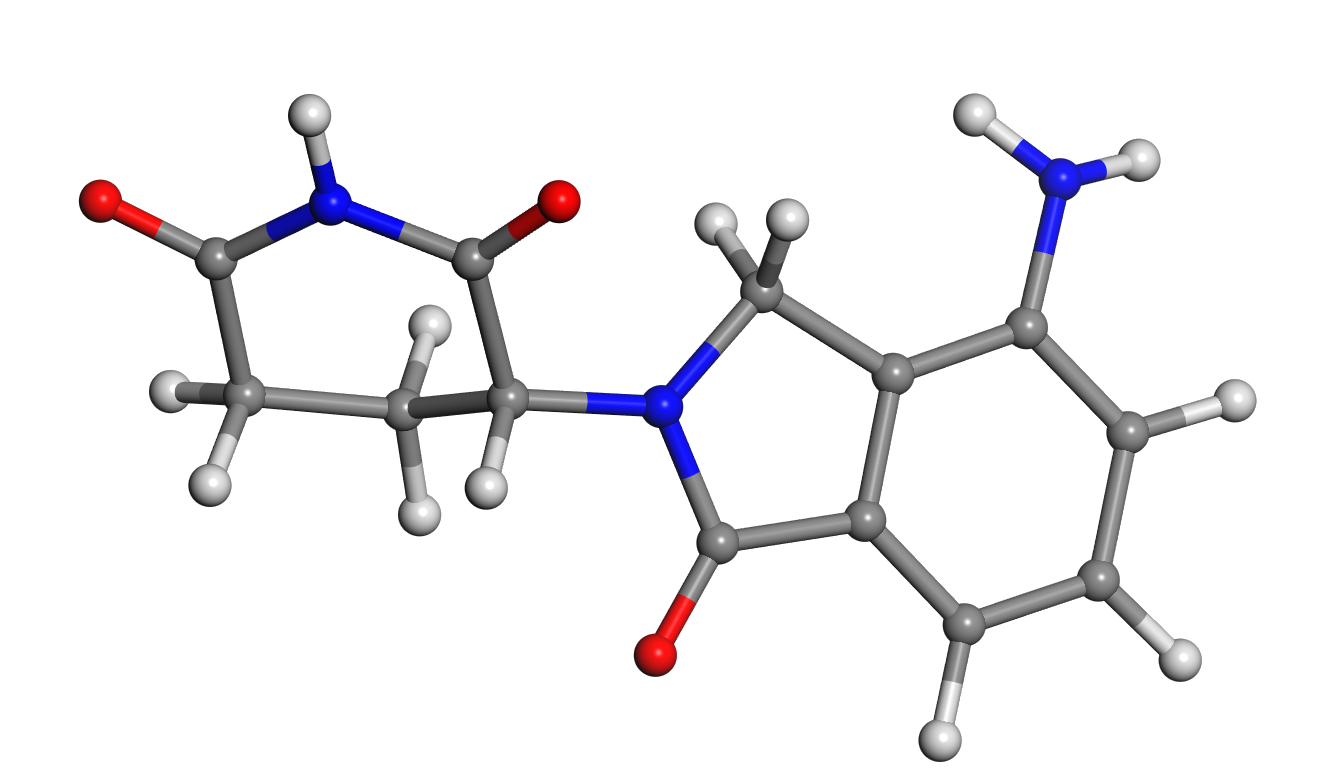
a


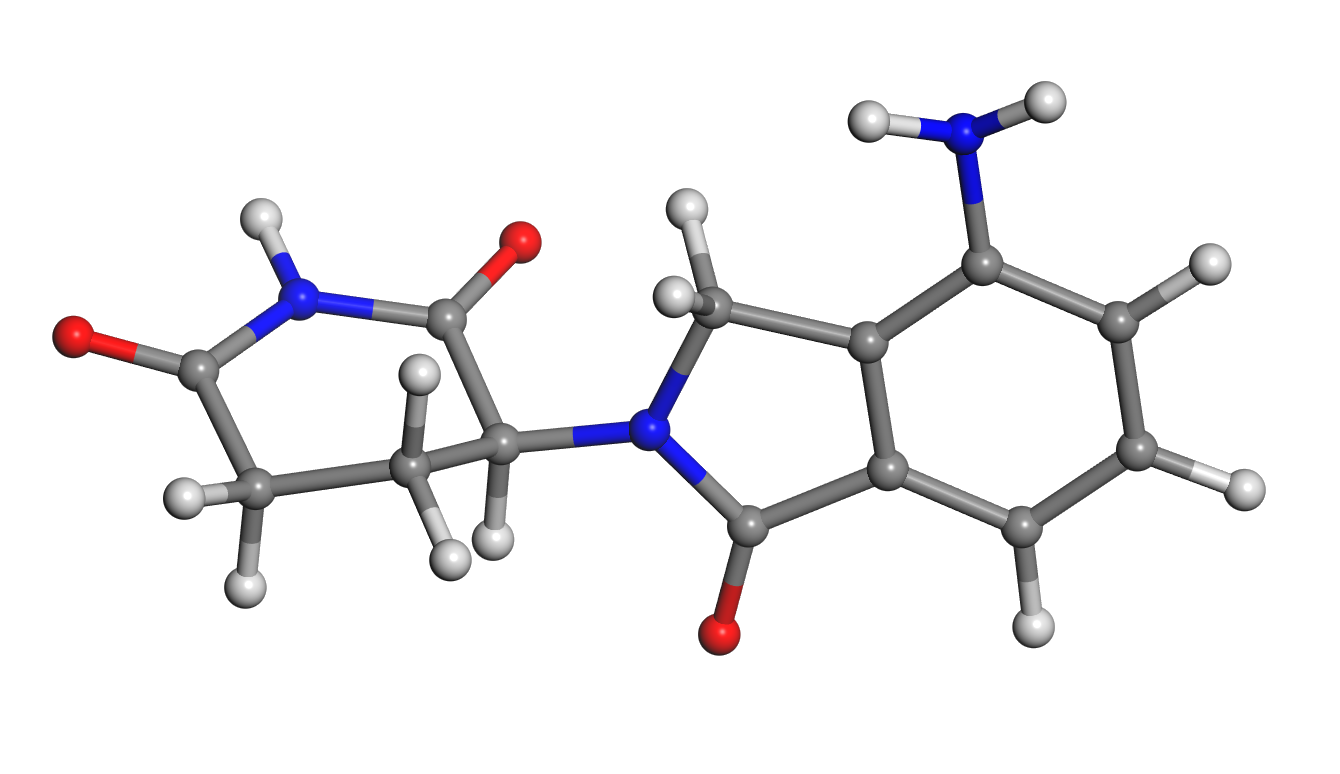
b

**Figure S1**. Molecular structure of LEN optimized in 3-D PBC with INTERFACE (a) and Dmol^3^ (b).

**Movie S1**: Molecular dynamics simulations of MNT-LEN complex with INTERFACE (MNT_Len_DMmovie file)

**Table S1** IR frequencies (in cm^-1^) calculated for the optimized (INTERFACE FF) structures comparing with experimental values

| Mode*^a^* | exp | molecule | LEN-1 | LEN-4 | MNT-LEN |
| --- | --- | --- | --- | --- | --- |
| ν(OH)_w_ |  |  |  |  | 3630-3416 |
| ν(OH)_oct_ |  |  |  |  | 3517-3511 |
| ν(NH_2_)*_as_* | 3475-3310*^b^*, 3407*^k^*, 3455*^m^* | 3529 | 3437-3435 | 3461-3458 | 3377 |
| ν(NH_2_)*_s_* | 3342*^k^*, 3362*^m^* | 3420 | 3337-3330 | 3341 | 3288 |
| ν(NH) | 3250-3140*^b^*, 3080*^k^* | 3497 | 3420-3415 | 3380 | 3454 |
| ν(CH) | 3080-*3050^b,c-d^,* 3185-3148*^k^*, 3080-3153*^m^* | 3048*^c^*, 3075*^d^*, 3066*^e^*, | 3096-3092*^e^*, 3080*^d^*, 3063*^c^* | 3082*^c^*, 3064*^d^*, 3096*^e^* | 3053*^c^*, 3075*^e^*, 3066*^d^* |
| ν(CH_2_) | 2975-2840*^b^*, 2964*^k^*, 3057*^m^* | 2984*^h^*, 2943*^f^*,  2938*^g^*, | 3007*^f^*, 2992*^h^*, 2940*^g^*, 2951*^f^*, 2941*^h^* | 2955*^f^*, 2965*^h^*, 3050*^h^*, 2970*^g^* | 3012*^h^*, 3005*^g^*, 2982*^f^*, |
| ν(CH_2_)*_s_* | 2964-2910*^k^* | 2899*^f^*, 2927*^h^*, 2885*^g^* | 2939*^h^*, 2893*^g^* | 2916*^g^*, 2913*^f^* | 2918*^f^*, 2904*^g^*,  2960*^h^* |
| ν(CH) | 2910-2879*^k^*, 2841*^m^* | 2924 | 2981 | 2949 | 2910 |
| ν(C=O) | 1740-1620*^b^*, 1700-1640*^k^*, 1702-1675*^m^* | 1801*^i^*, 1766*^f^*, 1790*^g^* | 1800*^i^*, 1772*^f^*, 1787*^g^* | 1797*^i^*, 1762*^f^*, 1792*^g^* | 1791*^g^*, 1785*^i^*, 1768*^f^* |
| δ(OH_2_)*_s_* |  |  |  |  | 1730-1673 |
| δ(NH_2_)*_s_* | 1630-1603*^k^*, 1633-1607*^m^* | 1698-1648 | 1698-1648 | 1720, 1709 | 1652 |
| ν(C=C)_rn_ | 1610-1400*^b^* | 1757, 1693 | 1761, 1695 | 1765, 1698 | 1759 |
| δ(CH) | 1486*^k^*, 1493*^m^* | 1542-1519*^c-d^* | 1571-1526*^c-d^* | 1545*^c-d^* |  |
| δ(CH_2_)*_s_* | 1480*^k^*, 1445*^m^* | 1481*^g^*, 1477*^f^*, 1464*^h^* | 1496*^f^*, 1506*^g^*, 1474*^h^*, 1495*^g^*, 1489*^f^* | 1486*^f^*, 1526*^g^*, 1467*^h^* | 1488-1443*^g^*, 1479*^f^*, 1463*^h^* |
| δ(NH) | 1460-1411*^k^*, 1445*^m^* | 1381 | 1441, 1407 | 1429-1425 |  |
| δ(CH) | 1341-1241*^k^*, 1350-1169*^m^* | 1352-1153 |  |  | 1534, 1476 |
| γ(CH) | 880*^k^*, 879*^m^* | 923 | 942-879 | 960-900 |  |

*^a^* Notation: *s* = symmetrical; *as* = asymmetrical; ν = stretching vibration; δ = bending in-plane vibration; γ = bending-out-of-plane vibration; w = water; oct = MOHM’ of octahedral sheet; rn = ring. *^b^* Interpreted by Chennuru et al. [14]. *^c^* Aromatic in meta position with respect to the amine group. *^d^* Aromatic in ortho position with respect to amine. *^e^* Aromatic in para position with respect to amine. *^f^* Piperidine ring in position α with the aromatic moiety. *^g^* In the heterocyclic ring. *^h^* Piperidine ring in position β with the aromatic moiety. *^i^* Piperidine ring in position γ with the aromatic moiety. *^j^* O=C-N-C=O. *^k^* Extracted from the spectra of LEN-1 [15]. *^m^* Extracted from the spectra of LEN-4 [14]

**Table S2** IR frequencies (in cm^-1^) calculated from the optimized (CASTEP) structures comparing with experimental values

| Mode*^a^* | exp | molecule | LEN-1 | LEN-4 |
| --- | --- | --- | --- | --- |
| ν(NH_2_)*_as_* | 3475-3310*^b^*, 3407*^k^*, 3455*^m^* | 3554 | 3455 | 3493-1490 |
| ν(NH_2_)*_s_* | 3342*^k^*, 3362*^m^* | 3445 | 3361 | 3363-3362 |
| ν(NH) | 3250-3140*^b^*, 3080*^k^* | 3425 | 3005 | 3052-3047 |
| ν(CH) | 3080-*3050^b,c-d^,* 3185-3148*^k^*, 3153*^m^* | 3115*^e^*, 3094*^c^*, 3074*^d^*, | 3114*^e^*, 3100*^c^*, 3088*^d^*, | 3117*^e^*, 3106*^c^*, 3092*^d^*, |
| ν(CH_2_) | 2975-2840*^b^*, 2964*^k^*, 3057*^m^* | 3026*^h^*, 3012*^f^*,  2919*^g^*, | 3048*^f^*, 3021*^h^*, 2940*^g^* | 3034*^h^*, 3026*^f^*, 2952*^g^*, 2946*^f^*ax, 2905*^h^*ax |
| ν(CH_2_)*_s_* | 2964*^k^* | 2949*^f^*, 2937*^h^*, 2877*^g^* | 2991*^f^*, 2942*^h^*, 2902*^g^* | 2915*^g^*, |
| ν(CH) | 2910-2879*^k^*, 2841*^m^* | 2909 | 2941-2939 | 2918 |
| ν(C=O) | 1740-1620*^b^*, 1700-1640*^k^*, 1702-1633*^m^* | 1721*^i^*, 1710*^f^*, 1697*^g^* | 1686*_s_^f,i^*, 1667*_as_^f,i^*, 1639*^g^* | 1692*^i^*, 1685*_s_^f,i^*, 1684*^f^*, 1650-1646*^g^* |
| δ(NH_2_)*_s_* | 1630-1603*^k^*, 1633-1607*^m^* | 1614, 1591 | 1630 | 1625-1595 |
| ν(C=C)_rn_ | 1610-1400*^b^* | 1598 | 1595-1587 | 1585 |
| δ(CH) | 1486*^k^*, 1493*^m^* | 1477-1443*^c-d^*, 1384*^c-d^* | (1475, 1307)*^c-d^* | 1485*^c-d^* |
| δ(CH_2_)*_s_* | 1460*^k^*, 1445-1418*^m^* | 1428*^g^*, 1439*^f^*, 1402*^h^* | 1452*^f^*, 1429*^g^*, 1383*^h^* | 1448-1435*^f^*, 1427-1425*^g^*, 1414*^h^*, 1393*^g^* |
| δ(NH) | 1460-1411*^k^*, 1675-1445*^m^* | 1366 | 1467 | 1675-1663 |
| δ(CH) | 1341-1241*^k^*, 1350-1169*^m^* | 1369, 1324-1143 | 1442, 1378, 1348-1154 | 1382-1275 |
| γ(NH) |  | 736 | 942 | 921 |
| γ(CH) | 880*^k^*, 983-879*^m^* | 790-736 | 947 | 982 |

*^a^* Notation: *s* = symmetrical; *as* = asymmetrical; ν = stretching vibration; δ = bending in-plane vibration; γ = bending-out-of-plane vibration; rn = ring. *^b^* Interpreted by Chennuru et al. [14]. *^c^* Aromatic in meta position with respect to the amine group. *^d^* Aromatic in ortho position with respect to amine. *^e^* Aromatic in para position with respect to amine. *^f^* Piperidine ring in position α with the aromatic moiety. *^g^* In the heterocyclic ring. *^h^* Piperidine ring in position β with the aromatic moiety. *^i^* Piperidine ring in position γ with the aromatic moiety. *^j^* O=C-N-C=O. *^k^* Extracted from the spectra of LEN-1 [15]. *^m^* Extracted from the spectra of LEN-4 [14].
